# Sonotubometry, a useful tool for the evaluation of the Eustachian tube ventilatory function

**Published:** 2014

**Authors:** A Borangiu, CR Popescu, VL Purcarea

**Affiliations:** * ”Carol Davila” University of Medicine and Pharmacy, Bucharest, “M.S. Curie” Clinical Hospital; ** ”Carol Davila” University of Medicine and Pharmacy, Bucharest, “Coltea” Clinical Hospital; *** Department 3, ”Carol Davila” University of Medicine and Pharmacy, Bucharest

**Keywords:** sonotubometry, Eustachian tube, ventilatory function, Digital Signal Processing

## Abstract

From the three Eustachian tube (ET) functions: middle ear protection, secretion clearance and middle ear ventilation, the ventilatory function is unanimously considered the most important one, because proper hearing is established only when tympanic membrane compliance is normal. This requires equilibrium between the middle ear and ambient gas pressure, which makes the normal functioning of active ET opening of critical importance. There are several methods and tests that can assess such a complex and variable mechanism. Sonotubometry is one such method; despite the fact that it has been continuously improved in the last 20 years, it is not yet systematically used to evaluate the ET ventilatory function, because its measurement pattern, context mapping (patient, clinic data, medication, treatment), validation, reproducibility and value for clinic practice, have not yet been fully consolidated and integrated in a knowledge-based, service-oriented system, that can provide decision support or even diagnostic. The paper reviews the role of tubal sonometry as a non-invasive, physiologic and easy to use method in assessing the ventilatory function and investigates the validity and reproducibility of a measuring pattern and test in a group of children. The paper describes the test pattern used, and the computer-based platform based on: (1) Digital Signal Processing (DSP) for sound acquisition and low-level processing; (2) Artificial Intelligence techniques to extract significant sound features from sonotubograms and learn a manifold context database. Results are reported from test series carried out in healthy children; a similar study between tests is included in the final Discussions section.

## Introduction

The Eustachian tube has three major physiologic functions:

• Middle ear protection from unwanted nasopharyngeal secretions and sound pressure.

• Clearance of middle ear secretions into the nasopharynx.

• Middle ear ventilation, providing pressure regulation, i.e. equilibrium of gas pressure in the middle ear relative to the atmospheric pressure.

The ventilatory function is assured by the regular and periodic opening of the ET. From these three physiologic functions, the most important is ventilation as it provides proper hearing at equilibrium of middle ear and ambient pressure; this happens at normal eardrum and middle ear compliance.

**Fig. 1:**
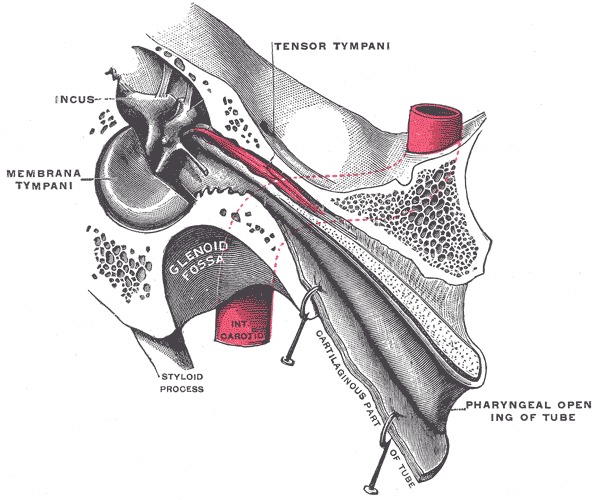
Eustachian tube anatomy (Gray’s Anatomy 20th edition, 1918)

Actually, the Eustachian tube is considered a component of a complete physiologic system consisting of the soft palate, nasal cavity, nasopharynx, middle ear and mastoid cavity (**Fig. 1**).

While normally the ET is passively closed, its intermittent active opening occurs when the tensor veli palatine muscle contracts during swallowing or yawning, causing ambient pressure in the middle ear nearby [**1**]. There is a certain spring-type action of the ET active muscular opening followed by the passive closing, which assures the clearance function of the tube [**2**].

ET function has been investigated in Malmö, Sweden; Bylander and colleagues proved that children have less efficient ET function than adults [**3**], although it does improve with aging.

For the pathogenesis of the middle ear diseases, dysfunctions of the ET have a most critical role. Other factors such as genetic, immunologic, infections, allergic and environmental factors are also involved; this explains why, due to structurally and functionally immature ET and not yet completely developed immune system there is a high incidence of otitis media in children [**4**].

The pathophysiology of the ET system has been studied over the past 40 years [**5**], and can be synthetized by tubes which: (a) will not open; (b) are too closed, too open, too floppy, too short or too stiff; (c) the system is either too closed or too open at either end of the ET; (d) there is abnormal pressure at either end of the system. The pressure regulation function of the middle ear mastoid can be hindered by an anatomic obstruction of the ET system, by a malfunction of the ET opening mechanism or by tube constricting during manoeuvres such as swallowing or yawning [**6**]. In children, opening failure it is quite common [**7**,**8**].

**Evolution of sonotubometry and other ventilatory function tests**

The opening of the Eustachian tube allows the ventilation of the middle ear cavity and the balancing of small pressure variations in the middle ear. In addition, there is continuous gas absorption through the blood vessels of the middle ear mucosa.

The measurements of the ET system’s ventilatory function have been subject of many clinical studies due to the high importance for clinical practice. Several types of tests have been, and are still used in research to assess the ventilatory function of the ET. An overview of the tests of pressure regulation function is given below.

**Classical tests**

In addition to the otoscopic investigation (also using a pneumatic attachment) allowing the visual inspection of the tympanic membrane, other tests of the ET pressure regulation function have been used; some of these tests are still used today:

1. *Valsalva test:* evaluates the effect of high positive nasopharyngeal pressures at ET’s terminal end; results are normal if the eardrum can be inflated by a forced expiration with closed nose.

2. *Politzer test:* consists in compressing one nostril into which a rubber tube attached to an airbag has been introduced while compressing the other nostril with finger pressure; the patient is asked to swallow. The result is normal when the overpressure created in the nasopharynx and transmitted via the middle ear creates positive middle ear pressure.

3. *Toynbee test:* the manoeuvre consists in swallowing when the nostrils are manually compressed, and thus a positive pressure phase is created in the nasopharynx, followed by a negative one. The test result is normal when there is a change in the middle ear pressure; e.g. a negative middle ear pressure or a temporary negative pressure alternating with normal middle ear gas pressure indicates a proper tubal function, meaning that ET opens actively [**9**].

**Tests used when the eardrum is intact**

**Fig. 2:**
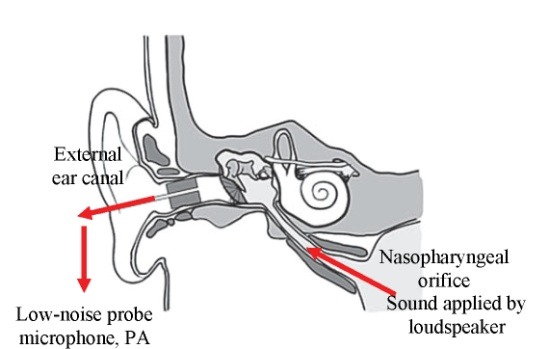
The sonotubometry method

The ET ventilatory function is evaluated by manometry, tympanometry or sonotubometry:

1. *Tympanometry:* consists in measuring the acoustic driving point immittance as function of the static pressure in the canal. By using low-frequency signals, the static pressure producing the maximal acoustic immittance is approximately equal to the gas pressure in the middle ear.

2. *Bluestone’s nine-step* test: this is an inflation-deflation test developed by Bluestone [**10**], which uses a tympanometer to evaluate the ET capacity to regulate pressure variations in the middle ear.

3. *Sonotubometry:* this type of test conveys sound from the nose to the Eustachian tube and captures it from the external ear (**Fig. 2**). A constant sound source (e.g. sine, 7 KHz, 100 dB) is applied to the nostril, while a microphone placed in the external auditory canal records the transmitted and alternated sound pressure through the Eustachian tube and middle ear. The patient performs a specific manoeuvre (swallowing, yawning, Valsalva) and in the normal case of ET opening, a significant increase in sound level will be registered in the external ear canal. Thus, the ventilatory function of the tube can be non-invasively evaluated, and the measurements occur under physiological circumstances, without the need to use external pressures or to perforate the tympanic membrane [**11**].

**Tests used when the eardrum is not intact**

The ET ventilatory function can be evaluated in such cases by using manometric measurements or sonotubometry:

1. *Modified Inflation-Deflation* test: the test is performed with the pump-manometer part of an electroacoustic immittance audiometer or a controlled syringe pump and manometer. During the test, sufficient positive pressure is applied to the middle ear to force the ET open (inflation). After the passive opening and closing, the pressure remaining in the middle ear (also defined as “residual positive pressure”) is registered and analysed [**12**,**13**].

2. *Sonotubometry:* This test is carried out exactly as in the case of intact eardrum; for a series of manoeuvres (swallowing, yawning, etc.) the number of tube openings is monitored, and sound features are extracted from sonotubograms both at test level (for the series of swallowing acts) and individual ET opening; these features are mapped to the manifold context (patient data) and integrated in a data base allowing patient differentiation and pathophysiological analysis.

**Material platform and method for sonotubometry tests**

**Signal processing for ET function evaluation**

Digital Signal Processing (DSP) techniques combined with data analytics in the stages of sound acquisition and low-level processing (noise rejection, variable filtering, monitoring alternation and feature extraction from the digital representation of the acoustic response signal to the stimulus during the opening time period of the ET) thus create the premises to confirm the validity and reproducibility of the best sequence and use these computed data for multi-criteria sonotubometry databases.

**Fig. 3:**
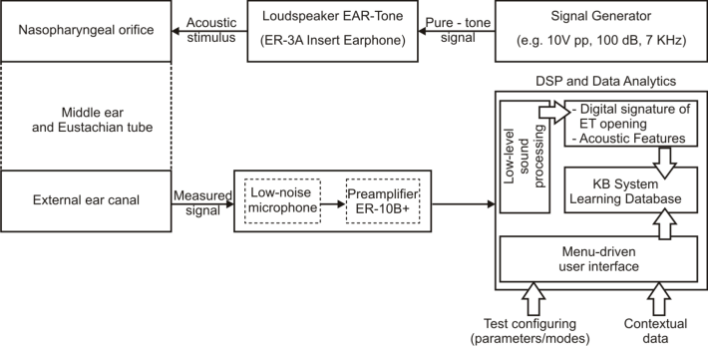
Computer-based platform for sound feature extraction and learning, manifold database tagging, ET function evaluation and utilization of a Knowledge Based system for decision support and diagnostic

A computer-based material platform for sonotubometry test has been developed to evaluate the ET function in children. This platform is designed and configured to allow sonotubometry tests both in healthy children and with middle ear pathology (serous otitis media, ET dysfunctions including cleft palate children), and operates in reconfigurable modes with the help of a menu-driven user interface. The data acquisition and analytics for low-level acoustic signal processing uses hardware and software DSP system integrated in a computer-based architecture (**Fig. 3**).

DSP performs sound acquisition and low-level acoustic signal processing. The software system has been designed to meet the following characteristics: (i) use pure-tone signals in the KHz range and adjustable acoustic intensity sufficiently high to support the attenuation caused by bad positioning, accidental dislocation of probes in the nose or ear, loss of probe airtightness or compression of nostrils; (ii) avoid the disturbing noise determined by pharyngeal activity (these signal components have frequencies lower than 5 KHz); (iii) eliminate noise pollution as much as possible caused by the ambient (especially high frequency components); (iv) disambiguate the information signal-response to the acoustic stimulus – from low amplitude noise generated by the mechanical activity of muscles, bones, epiglottis located close to the Eustachian tube (v) automatically detect the start of tube openings (from the amplitude and dynamics evaluation of the measured signal) and tag the corresponding moment of time on the filtered sound record; (vi) register significant changes in the sound intensity of the response to stimulus over the ET opening periods and create the “digital signature“ of the individual “tube opening shape diagram” (envelope of the sonotubogram); (vii) create a feature-based description of the “ET opening shape diagram”, extract and compute the numeric values of these sound features, and store them in a database for further high-level signal processing and interpretation; (viii) connect the DPS functions to an interactive user interface allowing the easy management of the measured sound records in term of: duration, disambiguation, parameter set-up, feature definition, context specification and proband data.

The pure sinusoidal stimulus signals were applied from an external signal generator to an ER-3A 50 Ohm Insert Earphone loudspeaker acting as sound source of 100 dB acoustic signal amplitude for the middle ear and Eustachian tube. This device provides external noise reduction of +30 dB.

The sonotubometry platform captures the sound transmitted through the ET from the external ear canal by using a low-noise ER-10 B+ microphone system, which includes:

• A microphone with foam tips to accommodate children ear canal size.

• A preamplifier (PA) powered by two 9V alkaline batteries with sensitivity of 50 mV/Pascal; the PA is switchable for 0, 20 or 40 dB additional low-noise gain computation.

• Standard front tubes of 0.95 mm OD x 0.58 mm ID x 76 mm length.

The sound captured by the microphone from the external auditory canal and amplified by the PA is input to a high-performance PC sound card installed on the PCI bus of a Lenovo ThinkPad T54OP laptop with Intel ® i7-700 MQ 2.4 GHz processor, 8 GB RAM and 500 GB SSD.

A LabView application for sound acquisition was created and installed in the low-level signal processing section of the software system developed for sonotubometry. This application is bi-directionally connected with the sonotubometry user interface to load configuring data for sound acquisition mode and parameters, and to store measured sound records and extracted features in the system’s storage and database.

The acoustic signal from the microphone is filtered by a software application to remove lower frequency components (generated by mechanical activities of noise generating sources located close to the ET: tongue, muscle and bones movements, epiglottis covering the windpipe) and higher frequency components (generated by the working environment), thus letting only the 7 KHz component of interest unaltered - response to input sound stimulus. The signal-filtering algorithm used in the reported tests can be schematized as it follows:

y(j)→fft(y)Y(k)→filtering [a,b]HzYf(k)→ifft(Y)yf(j)

where *j* is a time moment, *k* is a normed frequency and and *y_f (j)* is the filtered version of *y(j)*. This representation corresponds to the computation sequence:

Convert the measured signal-response to the sound stimulus from time representation vector to frequency representation, by applying the MATLAB Fourier transform function *fft(y)* (discrete Fourier transform of a vector of length N).

Remove the components having frequencies outside the band pass domain.

Compute the inverse transformation, from frequency domain to time domain, by using the MATLAB inverse function *ifft(y, N)* which calculates the inverse fast Fourier transformation of a vector of N length.

After filtering, sonotubograms (or “ET opening shape diagrams”) will be isolated from the 7 KHz non-zero component of the signal captured in the external auditory canal; each of these diagrams start at one ET opening, which is identified by a significant increase in the filtered sound intensity (greater than a predefined value threshold, e.g. 5-10 dB).

**The method. Eustachian tube opening manoeuvres**

For a sonotubometry test, an acoustic stimulus of frequency in the range from 6 KHz to 8 KHz and acoustic intensity between 100 dB and 120 dB is applied from a multifunctional signal generator via an ER-3A Insert Earphone loudspeaker to the nasopharyngeal ET ostium. A sinusoidal signal of 7 KHz frequency, 10 V amplitude peak to peak and 100 dB acoustic intensity have been used as input for the tests reported in this paper.

This input sound is conveyed through the ET to the middle ear and reaches the auditory canal. During induced tube openings (e.g. by swallowing), an increase in sound intensity level will be detected in the external ear canal. The signal from the ER-3A Insert Earphone is conveyed to the ET with the help of standard length plastic sound tube fixed to the child’s nostril by an air tightening foam eartip. These eartips are of medium and small size to fit to the children nostrils and have the same standard dimensions to ensure the proper calibration for the accuracy of test results and measured values. Thus, the length from the end of the eartip to the connection at the end of the earphone tube and the diameter have the values of 22 mm and 1.93 mm. Inter pharyngeal attenuation was reduced by the deep insertion of the foam tip in the nostril; after insertion, the foam expands and acoustically seals the nose canal from the environment.

Ambient noise reduction with the ER-3A earphone was typically of 30 dB in the frequency domain of 6-8 KHz used for tests. Audiometric air conduction testing down to 0 dB Hearing Level (HL) could be performed in the presence of background noise level lower than 45 dB.

The microphone system ER-10 B+ used in tests was inserted in the external auditory canal by means of medium- or small-sized foam tips for the same isolation purpose; it was connected through standard front tubes of 76 mm length and outer / inner diameter of 0.95 mm / 0.58 mm to a preamplifier (PA) switched for 20 dB additional low-noise gain. The acoustic signal from the microphone, amplified by the PA is the input to a PC sound card on the PCI bus, at a rate of 9600 samples/second; the sound card was configured for 1 channel, 16 bits resolution of quantisation, acquisition time / test of 10 seconds, and signal sample rate of 40 KHz.

Two types of noises that should be subtracted from the filtered 7 KHz information signal acquired from the auditory canal during tests (these sound levels are measured during the configuring stage of the sonotubometry material platform) have been defined:

*• Passive sound (baseline)* the sound level in the external ear canal measured when none of the ET opening manoeuvres were performed but the sound stimulus was continuously applied.

*• Background sound:* the level of noise generated by the environment with no sound stimulus.

In the tests performed in children and reported in this paper, ET tube openings were provoked by simple: (1) dry- and (2) liquid swallowing; the other forced manoeuvres will be used in future tests: (3) yawning; (4) Valsalva manoeuvre - closing the mouth, pinching the nose shut while pressing out in moderately forceful attempted exhalation against a closed airway; (5) Toynbee manoeuvre – swallowing with compressed nostrils.

The acoustic signals measured in the external ear were filtered and first processed in LabView environment by using Matlab low level DSP routines [**14**]; this data was stored in the PC in the form of digital records (sonotubograms) from which “ET opening shape diagrams” were created and stored as digital signatures. For the analysis scope, a number of sound features was defined and extracted from the ET opening shape diagrams to characterise the Eustachian tube openness in relation with the ventilatory function. At the sound acquisition and low level signal processing stage, a 5 dB variation in acoustic signal could be detected in an overall noise level of 70 dB intensity. Active and effective tube openings could thus be detected (*an active tube opening* must feature a maximum sound intensity of at least 20 dB, while at *an effective tube opening* the maximum sound intensity exceeds 30 dB over the passive sound level.

**Sonotubometry evaluation tests and results**

A number of 52 children, out of whom 25 girls and 27 boys aged 5–10 years (mean age: 6.4 years) considered otologically healthy were investigated in two sonotubometry tests, in subsequent days. The inclusion criterion was a negative history of ear pathology, including: acute otitis media, serous otitis media, ear complains, tympanic membrane trauma and hearing loss.

Prior to the sonotubometric testing, each child was evaluated in the ENT department of “M.S. Curie” Hospital for Children in Bucharest, according to the following investigation protocol: complete ENT examination focusing on the audiologic assessment: otoscopy (including pneumatic otoscopy), tympanometry (children with any other type of tympanometric curve than A were excluded), acumetry and pure tone audiometry. Only children without andenotonsillar, septal or cleft palate surgery, all having audiological tests in normal range, were included in these two tests. An informed consent was obtained from the children’s parents and clearance from the hospital’s ethical board.

The testing sessions took place in the audiology laboratory of “M.S. Curie” Hospital and consisted in 5 series of measurements (2 series per day) for each child: (1) first, the baseline (passive) sound intensity level was measured in the tested ear (the sound intensity registered in the ear canal with the signal generator turned on and delivered into the ipsilateral nostril, but without swallowing); (2) first each child performed a sequence of 6 consecutive dry swallowing acts, separated by a variable interval of a few seconds between each of them (this time interval varied inter- and intra- individually), followed by a few minutes pause and then a sequence of 6 water swallowing acts with the same methodology (sound intensity peaks were evaluated relative to the baseline intensity level); (2) theses 2 sequences of 6 dry and liquid swallowing acts were repeated in a subsequent day according to the same pattern, in order to investigate the reproducibility.

**Fig. 4:**
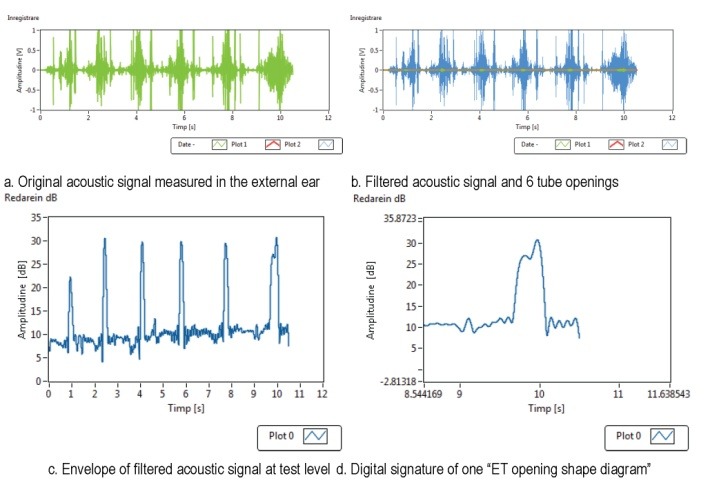
Measured acoustic signal and sonotubograms at test (global) and single ET opening (local) levels

**Fig. 4** shows the records for the measured acoustic signal at test level (a series of 6 dry swallowing acts) – the original acoustic signal measured in the external ear canal and its filtered version, and sonotubograms at test level (6 ET openings out of 6 swallowing acts) and for a single ET opening.

In order to minimize the sound transmission and capture variations (caused by possible dislocation of the nasal and ear foam tips), both the baseline sound intensity level AND the intensity level in the ear canal during the actual swallowing sequence were recorded without changing the position of the nasal and ear foam tips, and always after a thorough cleansing of nasal secretions and ear canal cerumen. We considered this measuring pattern to be important with respect to possible sound attenuations in the nasopharyngeal system determined by nasal turbinates, nasal secretions and/or nasal septal spurs. We randomised the chosen ear for testing in each child; however, the tests performed in the subsequent day were always performed in the same ear for a specific child.

The goal of these tests was to record and analyse the ET openings for each child in the various settings, the possible result being between 0 and 6 ET openings. An ET opening was deemed to be detected when the signal recorded in the tested ear canal showed an intensity increase of more than 10 DB SPL with respect to the baseline level, while an active ET opening was identified by a maximum sound intensity of at least 20 dB over the baseline intensity level.

For the first test session we recorded at least 1 tubal opening in 44 out of 52 children for dry swallowing (84.61%) and in 45 children for liquid swallowing (86.53%) with a mean tube openings number of 2.77 for dry swallowing and 2.64 for liquid swallowing. In 8 children we could not identify a tubal opening in the dry swallowing sequence; the same thing was noticed in 7 children when they performed liquid swallowing.

Only 3 children managed to open the ET every time they swallowed (1 of them showed this pattern both for dry and liquid swallowing and another 2 showed 6 openings in dry or liquid swallowing respectively). In the majority of children, the number of tubal openings recorded was either 2 or 3 for each session of 6 swallowing acts (for either dry or liquid).

For the second test session, similar results were obtained: there were 46 out of 52 children (88.46 %) who managed to show at least 1 ET opening for dry swallowing, with a mean openings number of 2.54, respectively 46 children (88.46 %) who demonstrated ET openings with a mean of 2.78 for liquid swallowing.

**Fig. 5:**
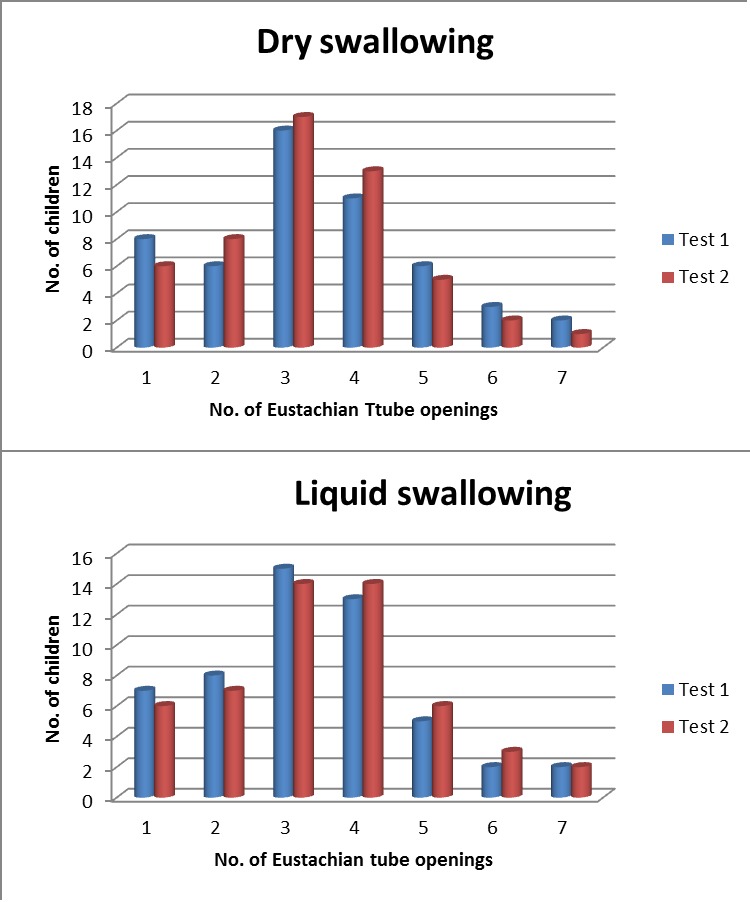
Comparative representation of the tests performed in 2 days for dry and liquid swallowing

As in the first session, there were 6 children who failed to demonstrate ET opening in dry swallowing, 6 for liquid swallowing, and 2 children who managed to open the ET every time they swallowed (but only one of them did it in both dry and liquid swallowing). In comparison, **Fig. 5** presents the results of the tests performed in two subsequent days for dry and liquid swallowing manoeuvres.

A similarity analysis has been done in order to evaluate the reproducibility of the method. The Euclidian distance between the results of measurements in the two days, expressed in active Eustachian tube openings, was computed separately for dry and water swallowing, then for the ensemble of the two opening manoeuvres:

Div=∑i=1ns(nciT1−nciT2)2∙nciT1+nciT2ncT1+ncT2

where: *nc_iT1*, *nc_iT2* are respectively the number of probands in Test 1 and Test 2 featuring i tube openings, 1≤*i*≤*ns*, *ns* = number of swallowing acts in one sequence for a certain procedure (dry swallowing or liquid swallowing). *Div* is the divergence between the two tests, for that procedure, indicating the unlikeness or non-similarity of the two tests.

For dry swallowing, the computed Euclidian distance is 1.56, for liquid swallowing 0.98 (which means a higher degree of similarity), whereas for the two global sonotubometry tests (dry and wet), the divergence is 1.31. The small values of these coefficients confirm the reproducibility of the method and measurement pattern.

## Discussions

The tests confirmed the role of sonotubometry as a valid and reproducible method for the registration in real time of the ET openings and physiological circumstances. At rest, a closed ET protects the middle ear from nasopharyngeal secretions and sound, whereas intermittent active opening reflects the tubal capacity to regulate the middle ear pressure variations.

The method is relatively easy to perform but involves a higher degree of patient cooperation than the tympanometry, which is widely used today to investigate the middle ear pressure changes.

Nevertheless, by using a thorough and patient approach, even young children (5 and 6 years old) were able to complete the sonotubometry test sequences. Considering the tested children’s history, free of any ear pathology, it appears to be of significance the fact that in every test sequence a significant number of children who failed to open the ET in all the swallowing sequences was recorded. This is consistent with some other findings by various investigators and the actual opinion that the ET is not opening with every deglutition and the gas pressure equilibrium is maintained through a combination of gas diffusion through the middle ear mucosa and gas transfer via the Eustachian tube.

There is a further need to address the specific question of predictive role of sonotubometry as an evaluation tool in the middle ear inflammatory pathology.

Another research direction aims at integrating the components necessary to build a knowledge-based system used in context-driven Eustachian tube function evaluation and decision support for otological diagnostic: feature-based modelling of digitized sound records; context-driven mapping of sound records in a manifold tagged sonotubometry knowledge base; iterative sound feature learning process; data analytics; predictive analysis for decision optimization. The computer-based sonotubometry platform will be further developed to provide two high-level functionalities:

• *Knowledge Base (KB) Feature Learning*: extracts features from individual and series of ET signal records (sonotubograms) and associates them to an operational context: patient data, clinic history, evolution of the health state, effects of medication and particularities of opening manoeuvres.

• *Decision Support*: once the KB created, it can be used for two important medical activities: (1) evaluation of the patient’s dynamic Eustachian tube function under physiological conditions, and (2) Real time otological diagnostic.

## References

[1] Honjo I, Okazaki N, Kumazawa T (1979). Experimental study of the Eustachian tube function with regard to its related muscles. Acta Otolaryngol.

[2] Bluestone CD (2005). Eustachian Tube, Structure, Function, Role in Otitis Media.

[3] Bylander A, Ivarsson A, Tjernström O (1981). Eustachian tube function in normal children and adults. Acta Otolaryngol.

[4] Kitajin M, Sando I, Hasjhida Y, Doyle W (1984). Histopathology of otitis media in infants with cleft and high arched palates. in: Lim DJ, Bluestone CD, Klein JO, Nelson JD, Eds., Recent advances in otitis media with effusion, Proceedings of the 3rd Int. Symposium.

[5] Bluestone CD, Berry QC (1976). Concepts on the pathogenesis of the middle ear effusions. Ann. Otol. Rhinol. Laryngol.

[6] Buchman CA, Doyle W, Skoner D (1994). Otologic manifestations of experimental rhinovirus infection. Laryngoscope.

[7] Swarts JD, Bluestone CD (2003). Eustachian tube in older children and adults with persistent otitis media. Int. J. Pediatr. Otorhinolaryngol.

[8] Van Heerbee N, Ingels KJ, Smile AF, Zielhuis GA (2001). Eustachian tube function in children after insertion of ventilation tubers. Ann. Otol. Rhinol. Laryngol.

[9] Elner A, Ingelstedt S, Ivarrson A (1971). The normal function of the Eustachian tube: a study of 102 cases. Acta Otolaryngol.

[10] Bluestone CD (1975). Assessment of Eustachian tube function. in: Jerger J. Ed., Handbook of clinical impedance audiometry. New York American Electromedics.

[11] Di Martino E, Thaden R, Krombach GA, Westhofen M (2004). Eustachian tube function tests current knowledge. HNO.

[12] Van der Avoort SJC, Van Heerbeek N, Zielhuis GA, Cremers WRJ (2005). Sonotubometry - Eustachian tube ventilatory function test; a review. Otol. and Neurology.

[13] Antweiler C, Telle A, Di Martino E, Vary P (2006). A New Otological Diagnostic System Providing a Virtual Tube Model.

[14] Borangiu A, Popescu D (2014). Digital Signal Processing for Knowledge-Based Sonotubometry of Eustachian tube function. Computer Eng. and Applied Informatics.

